# A Prospective Multicenter Study for Assessing MusiQoL Validity among Arabic-Speaking MS Patients Treated with Subcutaneous Interferon *β*-1a

**DOI:** 10.1155/2021/6681431

**Published:** 2021-03-02

**Authors:** Mohammed Al Jumah, Suleiman Kojan, Raed Alroughani, Edward Cupler, Saeed Bohlega, Abdulkader Daif, Mousa Al Mujalli, Talal Al Harbi, Mohamed El Tamawy, Samia Ashour, Chokri Mhiri, Riadh Gouider, Ayah Jawhary, Ahmed El Boghdady, Mohamed Hussein

**Affiliations:** ^1^King Fahad Medical City, MOH, As Sulimaniyah, Riyadh 12231, Saudi Arabia; ^2^King Abdullah International Medical Research Center, King Saud bin Abdulaziz University for Health Sciences, King Abdulaziz Medical City, National Guard Health Affairs, Riyadh 11481, Saudi Arabia; ^3^Division of Neurology, Department of Medicine, Amiri Hospital, Gulf Road, Bin Misbah St, Sharq, Kuwait; ^4^Department of Neuroscience, King Faisal Specialist Hospital and Research Centre, Al Rawdah Street, Jeddah, Saudi Arabia; ^5^Neurology Department, King Faisal Specialist Hospital, Zahrawi St, Riyadh 12713, Saudi Arabia; ^6^Neurology Department, King Khalid University Hospital, Riyadh 12372 6864, Saudi Arabia; ^7^Neurology Department, Herrah General Hospital, Makkah 20993, Saudi Arabia; ^8^Neurology Department, King Fahad Specialist Hospital, Ammar Bin Thabit St, Al Merikbat Neighborhood, Dammam 32253, Saudi Arabia; ^9^Neurology Department, Cairo University Hospital, Old Cairo, Cairo Governorate, Egypt; ^10^Neurology Department, Faculty of Medicine, Ain Shams University, El-Khalyfa El-Mamoun St, El-Abaseya, Egypt; ^11^Habib Bourguiba Hospital, Rue Al Firdaws, 3029 Sfax, Tunisia; ^12^Razi Hospital, 1st Street Ovanges, 2010 Manouba, Tunisia; ^13^Merck Serono, Tahaliyah St, 502 Jameel Square, Jeddah, Saudi Arabia, An Affiliate of Merck KGaA, Darmstadt, Germany

## Abstract

Few studies examine health-related quality of life (HRQoL) in Arabic-speaking multiple sclerosis (MS) patients. However, HRQoL tools such as the Short Form-36 QoL instrument (SF-36) and the Multiple Sclerosis International QoL (MusiQoL) questionnaire have been validated in other languages. The primary objective of this study was to prospectively assess HRQoL using the MusiQoL questionnaire among Arabic-speaking MS patients treated with subcutaneous interferon (sc IFN *β*-1a) over 12 months, as part of a prospective, multinational, multicenter cohort study. Patients' clinical parameters and HRQoL were assessed at baseline, 6 months, and 12 months. Changes in MusiQoL total and subdomain scores were compared using a Friedman test. Correlation between MusiQoL total score and Expanded Disability Status Score (EDSS) was also evaluated. In total, 439 patients from four Arabic-speaking countries were included. The mean age was 32.44 (±0.34) years, 71.5% were female, and 63.1% had an education level of university or above. The mean MS duration was 4.13 (±0.12) years, mean age at first attack was 27.35 (±0.26) years, and mean baseline EDSS score was 2.05 (±0.04). MusiQoL total score significantly improved at 6 months; however, this diminished at 12 months (65.67 ± 0.8 at baseline vs. 67.21 ± 0.79 at 6 months and 65.75 ± 0.8 at 12 months; *p* = 0.0015). Several aspects of patients' HRQoL including activity of daily living, physical well-being, symptoms, and coping improved. Overall HRQoL measured using SF-36 remained generally unchanged over time (*p* = 0.215). There was a statistically significant inverse relationship between change in EDSS score over time and change in overall MusiQoL score over time. In summary, findings confirm the utility of using MusiQoL for assessing changes in HRQoL during treatment with sc IFN *β*-1a in Arabic-speaking patients with MS.

## 1. Introduction

Professional interest among health care providers in the concept of health-related quality of life (HRQoL) has increased over the course of the past years, particularly within the framework of health care programs for the management of chronic diseases [1–4]. HRQoL measures can be subdivided into generic and disease-specific measures. Generic measures designed to assess patients with diverse medical conditions may not capture all relevant aspects of a specific illness. Furthermore, disease-specific measures developed from generic HRQoL tools may not truly reflect the perspectives from the patients about their specific disease.

Multiple sclerosis (MS), a chronic neurological disease, can have diverse effects on the lives of patients and their families. In controlled clinical trials, clinical measurement in MS has focused on impairments of neurological assessment using the Expanded Disability Status Scale (EDSS) [5]. Traditional clinical measures have not been able to assess the effects of neurological illness on quality of life, which is becoming an increasingly important topic to neurologists, with recommendations from MS experts advocating periodic assessment of HRQoL in the management of MS [1–3, 6].

The Multiple Sclerosis International Quality of Life (MusiQoL) questionnaire is a patient-focused instrument first developed in 2000 by an independent scientific steering committee in conjunction with MS patients, neurologists, and health economists. The questionnaire, which has since been validated in 14 languages and 20 countries [7], is a self-administered HRQoL indicator and is one of few globally specific MS HRQoL tools that reflect the patient's perceptions of their disease and its impact on their daily life. Furthermore, it is endorsed by the International MS Federation and has the advantage of being multidimensional (e.g., activity of daily living, psychological well-being, symptoms, relationships with friends, relationships with family, relationships with health care system, sentiment and sexual life, coping, and rejection) in its assessment [7]. The results of the MusiQoL validation study showed that the questionnaire presented a good acceptability (with less than 5% of missing items), as well as satisfying reliability and validity [7].

Our study was aimed at assessing HRQoL using the MusiQoL questionnaire in patients with relapsing MS treated with subcutaneous (sc) interferon *β*-1a (IFN *β*-1a; Rebif®) in Arabic-speaking countries and at assessing correlation between changes in HRQoL and clinical characteristics.

## 2. Materials and Methods

### 2.1. Study Design and Population

This was an observational, noninterventional, prospective, multicenter, multinational study including a total of 439 relapsing MS patients who were treated with sc IFN *β*-1a (44mcg) for at least 6 months prior to enrollment. Patients were recruited between January 2011 and January 2012 from governmental clinical centers in Saudi Arabia (*n* = 7), Kuwait (*n* = 1), Tunisia (*n* = 4), and Egypt (*n* = 2). At baseline (defined as the day on which the screening data were collected), demographic and clinical data were collected directly from eligible patients by self-administered questionnaires (MusiQoL and Short Form-36 Quality of Life instrument (SF-36)) and through neurological examination (including EDSS) and indirectly using the patient's medical charts (EDSS score, MS relapse assessment). The same measures and neurological examinations were performed on enrolled patients at 6 and 12 months, during which participating patients continued to receive sc IFN *β*-1a. Recall periods for the MusiQoL and SF-36 questionnaires were 4 weeks. Clinical charts and neurological examinations were used to collect demographic data, family history, paraclinical test results, treatment history, and medical events. The overall study duration from recruitment to treatment period was 2 years. Regarding MusiQoL, Arabic translation and linguistic validation were originally completed by the developer of the questionnaire (Mapi Research Trust, Lyon, France; https://eprovide.mapi-trust.org/instruments/multiple-sclerosis-international-quality-of-life-questionnaire).

Enrollment was limited to patients aged between 18 and 65 years. Additional inclusion criteria were a diagnosis of relapsing MS according to McDonald revised criteria [8]; an EDSS score ≤ 5; the ability to complete the MusiQoL; patients receiving treatment with sc IFN *β*-1a for 6 months prior to study initiation; and written informed consent.

Patients were excluded from the study if they met any of the following exclusion criteria: females who were pregnant and/or breastfeeding; females of childbearing age who were not using contraceptives; presence of other neurological conditions; and diagnosis of dementia or other major medical or psychiatric illness.

### 2.2. Study Endpoints

The primary endpoint was the change in HRQoL after 12 months, assessed by measuring the difference between MusiQoL total score at baseline and at 12 months. The secondary endpoints were the correlation between the MusiQoL total score and EDSS scores and the collection of additional data on MS patient quality of life using the SF-36 questionnaire.

### 2.3. Ethical Approval and Data Collection

Prior to commencement of the study at a given site, Independent Ethics Committee (IEC)/Institutional Review Board (IRB) approval was obtained. All eligible patients were informed of the study objectives, and inclusion/exclusion criteria were double-checked. The patients independently completed the questionnaires, usually in the waiting room prior to seeing the physician.

MS classification and ongoing MS treatments, including steroids, were recorded in the Case Report Form (CRF). A routine neurological examination, which included EDSS, was documented alongside the patient's relapse history. An MS relapse or exacerbation was defined as the appearance of a new symptom, or group of symptoms, or worsening/reappearance of an old symptom, attributable to MS, lasting at least 24 hours, in the absence of fever and preceded by stability or improvement for at least 30 days. The EDSS examination was performed by certified investigators, and where possible, the same investigator was used throughout the course of the study.

Any suspected adverse reactions that occurred during the study, whether serious or not, were captured in the CRF. A description of the reaction was recorded which included severity, duration (onset and resolution dates), causal relationship (i.e., confirmation that the adverse reaction is suspected to be reasonably related to the study treatment), any other potential causal factors, actions taken with the study drug (dose reduction, withdrawal), required treatment, and outcome. In the case of serious adverse reactions, seriousness criteria were documented and a specific safety form was completed and relayed to the investigators.

### 2.4. Quality of Life Measures

The MusiQoL questionnaire consists of 31 questions covering nine domains which include: (1) activity of daily living; (2) physical well-being; (3) relationships with friends; (4) symptoms; (5) relationships with family; (6) relationships with health care system; (7) sentimental and sexual life; (8) coping; and (9) rejection.

The SF-36 generic HRQoL instrument consists of 36 questions covering eight domains which include: (1) physical functioning; (2) role limitation due to physical health; (3) role limitations due to emotional problems; (4) energy/fatigue; (5) emotional well-being; (6) social functioning; (7) pain; and (8) general health.

### 2.5. Statistical Analysis

All demographic and clinical variables (at baseline) were summarized and reported using descriptive statistics. Interval variables such as age, age at first attack, MS duration in years, and EDSS score were summarized and reported in terms of mean and standard error. Categorical variables such as gender, city of residence, level of education, marital status, and type of MS and the presence of thyroid disease were summarized and reported as frequency distribution.

For MusiQoL, a score for each of the nine subdomains was calculated as the average of the scores for the set of questions making up the domain. An overall score was then calculated as the average of all the scores for each subdomain. If less than 50% of the answers were missing for any given subdomain, then the mean of the nonmissing answers was used to calculate the score for that domain. If more than 50% of the answers were missing, then the domain-specific score for that patient was kept as missing. Prior to computing the final overall score, each domain-specific score was linearly transformed to a 0-100 scale with zero being the worst quality of life and 100 the best.

For SF-36, an overall score was computed to represent the patient's overall quality of life as an average of all domain-specific scores. Domain-specific scores were calculated as the average score for all the questions comprising each specific domain. Unanswered questions were omitted from the calculation. As with MusiQoL, prior to computing the final overall score, each domain-specific score was linearly transformed to a 0-100 scale with zero being the worst quality of life and 100 the best.

Changes in the overall score for MusiQoL and SF-36, as well as for each subdomain, across all three time points (at baseline, 6 months, and 12 months) were analysed using the nonparametric Friedman test. To account for missing values in MusiQoL and SF-36 due to loss to follow-up, missing observations were imputed using the last-observation-carried-forward approach. All results were considered significant at type 1 error ≤ 0.05.

The relationship between change in quality of life over time and change in EDSS score over time was modeled using generalized estimating equation (GEE) regression. The repeated measurement of MusiQoL over time (at baseline, 6 months, and 12 months) was included as the dependent variable and EDSS score as a time-varying independent variable (at baseline, 6 months, and 12 months). A separate model was estimated for the overall score and for each domain. Correlations of change in MusiQoL and SF-36 and between change of MusiQoL, SF-36, and change in EDSS score were analysed using Pearson's correlation and its corresponding 95% confidence interval. All analyses were conducted using SAS version 9.2 (SAS Institute Inc., Cary, NC).

## 3. Results

A total of 439 patients treated with sc IFN *β*-1a for relapsing MS were included in the study. Of these, 299 were recruited from Saudi centers and the remaining 140 were recruited from international centers (including Kuwait, Tunisia, and Egypt). Mean patient age at baseline was 32.44 (±0.34) years. Around half of patients (53.5%) were married, and most had an educational level of university degree or higher (63.1%). Patients had a mean MS duration of 4.13 (±0.12) years, a mean EDSS score of 2.05 (±0.04), and a mean age at first attack of 27.34 (±0.26) years. In total, 87.6% of patients had no family history of MS, 77.8% had no history of family consanguinity, and 95.4% did not have any sibling affected with MS (Table 1). In general, patients recruited from Saudi centers had similar baseline characteristics to patients recruited from other centers except for some differences in patients' education and MS duration; patients from international centers were more likely to have had a university degree or above (74.1% vs. 56.6%; *p* = 0.001) and also had shorter average MS duration (3.25 vs. 4.65 years; *p* = 0.0001) (Table 1).

In all centers, the most common symptoms (occurring in at least 20% of patients) experienced at first MS attack included motor weakness (33.4%), sensory symptoms (40.5%), visual symptoms (38.2%), and ataxia (16.5%). Almost all patients (99.3%) in the study had one of their central nervous system (CNS) regions affected at first attack. Affected cranial nerve (CN) regions included visual pathway (35.2%), cerebellar region (23%), and sensory function region (27.1%).

### 3.1. Change in HRQoL

Of the 439 patients enrolled, some 384 (87.5%) had complete MusiQoL and SF-36 data at 6 months and 330 (75.2%) at 12 months. Missing data for MusiQoL and SF-36 were primarily due to dropouts as a result of medication discontinuation given adverse effects or lack of improvement. Findings for changes in MusiQoL and SF-36 scores are summarized below.

### 3.2. MusiQoL

Compared with baseline, MusiQoL total score was significantly increased at 6 months but regressed towards baseline at 12 months (baseline, 65.67 ± 0.8; 6 months, 67.21 ± 0.79; 12 months; 65.75 ± 0.8; *p* = 0.0015). Many of the MusiQoL subdomains including activity of daily living (*p* = 0.0011), physical well-being (*p* < 0.0001), symptoms (*p* = 0.0004), and coping (*p* = 0.0029) were significantly increased over time. On the other hand, sentimental and sexual life (*p* = 0.0072) and relationships with health care systems (*p* = 0.0424) showed statistically significant decreases (Table 2).

### 3.3. SF-36

Over time, change in quality of life (overall score) measured by SF-36 was not statistically significant (*p* = 0.215). Other than social functioning (*p* = 0.038), all other domains (general health, *p* = 0.8186; physical functioning, *p* = 0.4063; role limitation due to physical health, *p* = 0.379; role limitation due to emotional problems, *p* = 0.4828; energy/fatigue, *p* = 0.3158; emotional well-being, *p* = 0.3539; and pain, *p* = 0.2764) did not show a statistically significant change over time (Table 3).

### 3.4. Relationship between Change in EDSS Score and MusiQoL Over Time

There was a statistically significant inverse relationship between change in EDSS score over time and change in MusiQoL total score. One point increase in EDSS score was associated with 1.44 (*p* = 0.0003) point decrease in MusiQoL score. Change in EDSS score was also inversely related to change in MusiQoL domain scores, including activity of daily living (-3.53; *p* < 0.001), symptoms (-1.36; *p* = 0.01), relationships with health care system (-1.44; *p* = 0.003), coping (-2.08; *p* = 0.0028), and rejection (-2.47; *p* = 0.0003) (Table 4).

## 4. Discussion

The concept of HRQoL is defined by the World Health Organization as an individual's perception of their life within the cultural context and value system in which they live, in respect to objectives, expectations, norms, and worries. It is a complex and broad concept, influenced at various levels by the patient's physical health state, psychological health state, level of independence, social relations, and relationship with the overall surrounding environment. A critical element of HRQoL is that it reflects the patient's assessment of the impact of his/her illness, not the physician's perspective, as most physiologically oriented measures and traditional clinical scales do [1]. Faced with the limitations of conventional indicators which are unable to assess long-term health care management, health care professionals involved with care of patients with MS are increasingly focusing on evaluating the well-being of their patients using tools such as the MusiQoL questionnaire, which is the only globally specific MS HRQoL tool constructed from the MS patients' perspective [7].

The aim of the present study was to prospectively assess HRQoL in Arabic-speaking MS patients over 12 months using the MusiQoL questionnaire, in patients treated with sc IFN *β*-1a for at least 6 months. At baseline, the mean MusiQoL total score was 65.67 ± 0.8 demonstrating that overall, the patients' mostly assessed their quality of life positively. These positive perceptions of HRQoL were also observed in a MusiQoL study conducted in Lebanon [9].

In all sites, the MusiQoL total score showed statistically significant improvement from baseline to 6 months, primarily driven by improvements in activity of daily living, physical well-being, symptoms, and coping. We regard such improvement as clinically relevant, given the improvement across various domains and that the mean change in MusiQoL total score was generally consistent with a 1-point difference in EDSS score. However, the improvement in MusiQoL total score regressed towards baseline at 12 months due to deterioration in psychological factors, including statistically significant reductions in sentimental and sexual life and relationships with health care systems. There were also nonsignificant reductions in relationship with friends, relationship with family, and rejection. These findings are in line with the results of a bicenter study assessing HRQoL in MS patients from Northern Iran, which demonstrated that psychological factors such as depression, anxiety, and stress were key predictors of reduced quality of life [10].

In addition to MusiQoL, our study also made use of the generic SF-36 questionnaire to assess patient quality of life. Using both the MusiQoL and SF-36, similar overall assessments of quality of life were observed (i.e., a positive perception). However, although there was a statistically significant increase in quality of life measured using MusiQoL, the overall quality of life measured using SF-36 remained generally unchanged over time (*p* = 0.215), with only social functioning demonstrating any significant improvement (*p* = 0.038). These observations are likely explained by the fact that SF-36 is a generic instrument and is often not sensitive enough to pick up the subtle improvements in HRQoL detected by a disease-specific instrument, such as MusiQoL. Furthermore, if the two components of SF-36 are examined, differences can be seen, with patients viewing the mental aspects more positively than the physical aspects, while for the MusiQoL, the physical aspects were viewed more positively. These differences may be related to the responsiveness of each tool to measuring the different dimensions of quality of life that are important to patients with MS. A longitudinal study of MS patients carried out at 32 centers in 12 non-Arabic countries, for example, found that MusiQoL was more responsive to “nonphysical” dimensions, such as relationships with health care system and sentimental and sexual life, while the SF-36 was more adept at detecting changes in the “physical-like” dimensions (such as physical functioning and bodily pain) [11].

A notable finding of our study was that the patients' perception of their HRQoL, as determined by MusiQoL, corresponded to their neurological (disability) status. Indeed, there was a statistically significant inverse relationship between change in EDSS score over time and change in MusiQoL total score, with a 1-point increase in EDSS score associated with a 1.44 (*p* = 0.0003) point decrease in MusiQoL total score. These observations were similar to a study assessing HRQoL in the Middle East and North Africa region which found that a higher EDSS scores was associated with a lower MusiQoL score [9].

A potential limitation of this study was that approximately 25% of patients had a missing MusiQoL or SF-36 scores by the end of the study, which may have impacted on the results. Missing values were mainly due to lost to follow-up as a result of side effects of the medication or treatment switching due to lack of clinical efficacy. To control for missing data, we used the last-observation-carried-forward approach that is known to bias the results towards the null, thereby providing a conservative estimate of change in quality of life over time. However, a sensitivity analysis of patients with no missing values during follow-up did not alter the study findings. Another limitation is the lack of a control group, which made it difficult to judge whether the improvement in HRQoL was due to time alone, medication alone, or a combination of both. Moreover, to gain a better understanding of the factors impacting HRQoL, the study would have benefited from assessing the correlations between the MusiQoL total score and the following clinical characteristics at baseline and after 12 months of treatment: disease duration, number of relapses in the previous twelve months, and demographic characteristics. Despite such limitations, study strengths include the fact that it was completed as per protocol and the enrolled population was in accordance with the inclusion/exclusion criteria. The demographic and MS conditions were representative of the expected patient population. The changes in HRQoL assessed by MusiQoL were reflected in the observed clinical changes. The disease-specific sensitivity of MusiQoL was reconfirmed in this study. The total score was shown from calculation of effect size to be less informative of real change than an examination of the individual measured dimensions. One reason for this was that changes in component scores partially cancelled each other out as some were positive and some were negative.

## 5. Conclusion

Periodic assessment of quality of life is highly recommended in the management of MS patients. Overall, the findings of this study support the use of MusiQoL to evaluate quality of life among Arabic-speaking MS patients.

## Figures and Tables

**Table 1:** Key patients' baseline demographics and clinical characteristics.

Variable	Category	Overall (*N* = 439)	Saudi patients (*N* = 299)	Other Arabic countries (*N* = 140)	*p* value
Patient's gender	Female	71.5%	69.8%	74.3%	0.403
	Male	28.5%	30.2%	25.7%	
Patient's education	High school or less	36.9%	43.4%	25.9%	0.001
	University & above	63.1%	56.6%	74.1%	
Patient's marital status	Married	53.5%	53.7%	53.2%	1
	Single/divorced/widowed	46.5%	46.3%	46.8%	
Family history of MS	No	87.6%	85.3%	92.1%	0.06
	Yes	12.4%	14.7%	7.9%	
Parental consanguinity	No	77.8%	77.1%	79.1%	0.711
	Yes	22.2%	22.9%	20.9%	
Affected siblings	No	95.3%	94.4%	97.1%	0.328
	Yes	4.7%	5.6%	2.9%	
Patient's age	Mean ± S.E	32.44 ± 0.34	32.66 ± 0.76	31.81 ± 0.74	0.5265
Age of first attack	Mean ± S.E	27.35 ± 0.26	27.20 ± 0.5	27.13 ± 0.7	0.9809
MS duration (years)	Mean ± S.E	4.13 ± 0.12	4.65 ± 0.27	3.25 ± 0.29	0.0001
EDSS score	Mean ± S.E	2.05 ± 0.04	2.11 ± 0.08	1.97 ± 0.1	0.5913

EDSS: Expanded Disability Status Scale; MS: multiple sclerosis; S.E: standard error.

**Table 2:** Overall and domain-specific MusiQoL scores over time.

Variable	Initial visit (*N* = 439)	6-month follow-up (*N* = 439)	12-month follow-up (*N* = 439)	*p* value
MusiQoL total score	65.67 ± 0.8	67.21 ± 0.79	65.75 ± 0.8	0.0015
Activity of daily living	62.84 ± 1.31	64.92 ± 1.26	64.95 ± 1.21	0.0011
Physical well-being	55.47 ± 1.4	59.96 ± 1.37	61.02 ± 1.22	<0.0001
Symptoms	58.61 ± 1.17	60.45 ± 1.11	61.94 ± 1.12	0.0004
Relationship with friends	59.88 ± 1.38	59.87 ± 1.34	57.34 ± 1.29	0.1868
Relationship with family	74.15 ± 1.29	75.22 ± 1.23	71.17 ± 1.28	0.2341
Sentimental & sexual life	56.88 ± 1.55	56.38 ± 1.53	54.98 ± 1.44	0.0072
Coping	66.37 ± 1.47	69.95 ± 1.35	68.31 ± 1.31	0.0029
Rejection	76.72 ± 1.32	77.08 ± 1.3	75.50 ± 1.3	0.3679
Relationship with health care system	73.87 ± 1.05	74.44 ± 1.06	70.44 ± 1.13	0.0424

Values are mean ± standard error; *p* values correspond to the change in overall and domain-specific MusiQoL scores across all 3 times points analysed using the nonparametric Friedman test.

**Table 3:** Overall and domain-specific SF-36 scores over time.

Variable	Initial visit (N =439)	6-month follow-up (*N* = 439)	12-month follow-up (*N* = 439)	*p* value
SF-36 total score	56.86 ± 1.15	57.74 ± 1.13	57.84 ± 1.07	0.215
General health	51.94 ± 0.82	51.24 ± 0.8	51.97 ± 0.78	0.8186
Physical functioning	66.23 ± 1.33	67.39 ± 1.3	65.14 ± 1.31	0.4063
Role limitations due to physical health	53.19 ± 2.06	54.21 ± 2.08	54.29 ± 2.03	0.379
Role limitations due to emotional problems	55.56 ± 2.14	55.58 ± 2.12	58.11 ± 2.09	0.4828
Energy/fatigue	49.41 ± 1.04	50.40 ± 0.99	51.22 ± 0.94	0.3158
Emotional well-being	58.50 ± 1.05	60.17 ± 0.94	59.57 ± 0.9	0.3539
Social functioning	66.03 ± 1.32	67.63 ± 1.24	67.17 ± 1.22	0.038
Pain	57.70 ± 1.11	58.77 ± 1.07	58.79 ± 1.01	0.2764

Values are mean ± standard error; *p* values correspond to the change in overall and domain-specific MusiQoL scores across all 3 times points analysed using the nonparametric Friedman test.

**Table 4:** Relationship between change in EDSS score and change in overall and domain-specific MusiQoL scores over time.

	EDSS score				
	Estimate	Standard error	95% lower confidence limit	95% upper confidence limit	*p* value
MusiQoL total score	-1.4415	0.4027	-2.2308	-0.6521	0.0003
Activity of daily living	-3.528	0.5913	-4.687	-2.3691	<0.0001
Physical well-being	-0.8058	0.6675	-2.1141	0.5025	0.2273
Symptoms	-1.3574	0.5268	-2.3898	-0.3249	0.01
Relationship with friends	-0.5383	0.7188	-1.947	0.8705	0.4539
Relationship with family	-0.938	0.6882	-2.2868	0.4108	0.1729
Sentimental & sexual life	-1.1681	0.747	-2.6323	0.296	0.1179
Coping	-2.0795	0.6945	-3.4406	-0.7183	0.0028
Rejection	-2.4697	0.685	-3.8123	-1.1271	0.0003
Relationship with health care system	-1.4415	0.4027	-2.2308	-0.6521	0.0003

The relationship between EDSS and MUSIQOL was analysed using generalized estimating equation (GEE) regression; *p* values correspond to the significance of the trend over time of both variables.

## Data Availability

Any requests for data by qualified scientific and medical researchers for legitimate research purposes will be subject to Merck KGaA's Data Sharing Policy. All requests should be submitted in writing to Merck KGaA's data sharing portal https://www.merckgroup.com/en/research/our-approach-to-research-and-development/healthcare/clinical-trials/commitment-responsible-data-sharing.html. When Merck KGaA has a coresearch, codevelopment, or comarketing or copromotion agreement, or when the product has been out-licensed, the responsibility for disclosure might be dependent on the agreement between parties. Under these circumstances, Merck KGaA will endeavour to gain agreement to share data in response to requests.
